# Comparison of postoperative survival prognosis between early-onset and late-onset esophageal cancer: A Population-based study

**DOI:** 10.1371/journal.pone.0315391

**Published:** 2024-12-12

**Authors:** Lang Qin, Jie Tian, Yuan Zhang, Yanlin Yin, Zhenling Dou, Jie Chen, Zhi Zhang, Yu Gong, Wenhua Fu

**Affiliations:** 1 Department of Radiotherapy, Huainan Chaoyang Hospital, Huainan, China; 2 Department of Oncology, Huainan Chaoyang Hospital, Huainan, China; Universitá Sapienza di Roma, ITALY

## Abstract

The prognosis of non-distant metastatic early-onset esophageal cancer (EC) patients undergoing surgical treatment remains unclear, this study aims to compare the prognosis of early-onset and late-onset EC. Information on non-distant metastatic EC patients who underwent surgical treatment and were initially diagnosed between 2004 and 2015 was collected from the Surveillance, Epidemiology, and End Results (SEER) database. Propensity score matching (PSM) was used to balance the baseline differences between early-onset and late-onset EC patients. Univariate and multivariate Cox regression analyses were used to calculate hazard ratio (HR) and 95% confidence interval (CI). The Kaplan-Meier method and log-rank test were used to compare the overall survival (OS) between the two groups of patients. Atotal of 5320 EC patients were included, with 571 in the early-onset group and 4749 in the late-onset group. Multivariate Cox regression analysis showed that early-onset EC patients had better OS (HR = 0.732, 95% CI: 0.655–0.819, p<0.001). Using PSM analysis at a 1:1 ratio, we matched 557 early-onset EC patients with 557 late-onset EC patients. After matching, the multivariate Cox regression model still showed a favorable prognosis for early-onset EC (HR = 0.728, 95% CI: 0.630–0.842, p<0.001). Additionally, subgroup analysis indicated that early-onset EC patients had better long-term prognosis. Non-distant metastatic early-onset EC patients undergoing surgical treatment demonstrated better OS outcomes, confirmed by regression analysis and subgroup analysis in the matched cohort.

## Introduction

Esophageal cancer (EC) is one of the leading causes of cancer mortality worldwide, ranking seventh in incidence and sixth in mortality, it is estimated that in 2020, 1 in every 18 cancer deaths was due to EC [1]. The two most common histological subtypes of EC are esophageal squamous cell carcinoma (ESCC) and esophageal adenocarcinoma (EAC), ESCC is more prevalent in Asian populations, with alcohol consumption and smoking being significant risk factors, while EAC is more common in Caucasians and is closely associated with obesity and smoking [2, 3]. Currently, surgery is considered one of the most important treatments for EC. Despite significant advancements in modern surgical techniques, radiotherapy, and chemotherapy, the 5-year overall survival (OS) rate after surgery has gradually improved but remains below 40% [4, 5].

EC can be classified into early-onset EC and late-onset EC based on the age of onset. Most studies define early-onset EC as a malignant tumor diagnosed before the age of 50 [6–9]. According to international reports, the incidence of malignant tumors in young people, including early-onset EC, has been on the rise globally, since the 1990s, the incidence of early-onset EC in the United States has increased by 50% [10, 11]. However, whether prognosis differs between early-onset and late-onset EC is still debated. A Dutch study reported that although early-onset EC had higher rates of lymph node positivity and distant metastasis, the proportion of patients undergoing surgery was higher compared to older patients, and there was no significant difference in 5-year OS rates [12]. Markar et al study found that early-onset EC patients undergoing esophagectomy had similar staging to late-onset EC, fewer postoperative complications, but no significant difference in OS [13]. Kolb et al found that early-onset EAC had more advanced staging but higher survival rates compared to older patients. However, they did not further analyze whether the differences in survival were due to variations in treatment methods [14].

The factors influencing the survival differences between early-onset and late-onset EC are multifaceted. Compared to late-onset EC, early-onset EC may present at a more advanced stage. Still, patients with early-onset EC typically have better overall health and can tolerate more aggressive treatment regimens [14, 15]. Previous studies included patients with significant baseline differences and the survival differences between early-onset and late-onset EC patients were influenced not only by age but also by confounding factors such as tumor stage and treatment modalities, raising questions about the conclusions [12–14]. Considering that the current treatment for EC is primarily surgical, we used propensity score matching (PSM) to adjust for potential confounding factors and specifically analyze the survival differences between early-onset and late-onset non-distant metastatic EC patients who underwent surgical treatment.

## Methods

### Patient selection and study variables

The SEER (http://seer.cancer.gov/seerstat/) database is an important cancer data resource maintained by the National Cancer Institute (NCI) of the United States. The SEER database covers approximately 34.6% of the population and represents a wide distribution of different racial, gender, and age groups nationwide. It collects and provides detailed information on cancer incidence and survival rates in the U.S. population. Data can be accessed online using SEER*Stat software version 8.4.0.1.

Inclusion criteria include: (1) Clear pathological diagnosis of esophageal tumor location; (2) First primary tumor; (3) Initial diagnosis between 2004 and 2015; (4) Received surgical treatment. Exclusion criteria include: (1) Demographic characteristics such as age, race, and unknown marital status; (2) Clinical and pathological characteristics such as unknown primary tumor site, tumor size, histological type, grade, and stage; (3) Treatment information including unknown surgery, radiotherapy, and chemotherapy.

The risk factors included in the analysis are demographic characteristics such as age, sex, race, and marital status; tumor characteristics such as histological type, primary tumor site, tumor length, differentiation grade, T stage, and N stage; treatment information such as surgery, radiotherapy, and chemotherapy; and follow-up data such as survival status and survival time. The study endpoint is OS, defined as the time from the beginning of the study until death from any cause. To obtain reliable follow-up data, patients included were diagnosed between 2004 and 2015, with the last follow-up conducted in November 2020, ensuring a minimum follow-up of five years for patients still alive at entry. For histological type, ICD-O-3 codes 8140–8145, 8210, 8211, 8255, 8260, 8261, 8263, 8310, 8480, 8481, 8490, 8574 are defined as EAC; ICD-O-3 codes 8050–8052, 8070–8076, 8083–8084 are defined as ESCC, and all other codes are defined as other types. For tumor site, "Cervical esophagus" and "Upper third of esophagus" are defined as upper segment, "Middle third of esophagus" is defined as middle segment, "Lower third of esophagus" and "Abdominal esophagus" are defined as lower segment, "Overlapping lesion of esophagus" and patients with unspecified tumor site "Esophagus, NOS" and "Thoracic esophagus" are defined as other.

### PSM

PSM is a method used to address distribution bias in observational data, aiming to reduce bias caused by confounding factors in observational studies and emulate the effects of randomized controlled trials. PSM calculates a propensity score for each participant to receive a specific treatment or exposure, then matches participants with similar scores to balance confounding factors between groups. By using PSM, differences in variables other than age between early-onset EC and late-onset EC are eliminated, focusing observation on the impact of age on long-term prognosis, and minimizing bias in statistical analysis caused by confounding factors, thereby enhancing the reliability of the final results.

### Statistical analysis

Pearson’s chi-square test was used to analyze differences in variable distribution between early-onset EC patients and late-onset EC patients. PSM was used to match early-onset EC patients and late-onset EC patients in a 1:1 ratio. The univariate and multivariate Cox regression analysis were used to examine the relationship between variables and OS, and calculate hazard ratios (HR) and 95% confidence intervals (CI). Kaplan-Meier method was used to visually analyze the OS of different subgroups of patients, and the presence of statistical differences was assessed using the log-rank test. A p-value < 0.05 is considered to indicate statistical significance.

X-tile 3.6.1 (Yale University School of Medicine, USA) was used to determine the optimal cutoff value for tumor size. Statistical analyses in this study were performed using R statistical software (version 4.2.1, http://www.R-project.org). Specifically, the "Matching" package and "tableone" package were used for PSM; the "rio" package was used for data output; the "survival" package and "survminer" package were used for Kaplan-Meier survival curve plotting and log-rank test; the "survival" package, "forestplot" package, and "survminer" package were used for univariate and multivariate Cox regression analysis.

## Results

### Patient characteristics

A total of 5,320 patients with EC who underwent surgical treatment between 2004 and 2015 were evaluated, with 571 patients in the early-onset EC group and 4,749 patients in the late-onset EC group (Table 1). Before PSM, significant differences (p<0.05) were observed between the two groups in terms of race, marital status, primary tumor site, tumor size, T stage, N stage, chemotherapy, and radiotherapy. Specifically, compared to late-onset EC patients, early-onset EC patients had a higher proportion of Black individuals (7.53% vs. 5.01%, p = 0.001) and a lower proportion of White individuals (85.29% vs. 90.38%, p = 0.001). They also had a higher proportion of unmarried individuals (39.75% vs. 31.65%, p<0.001), more tumors located in the lower esophagus (81.44% vs. 75.70%, p = 0.001), more tumors larger than 50 mm (30.82% vs. 24.32%, p<0.001), higher proportions of T3 (50.79% vs. 57.53%, p<0.001) and T4 stages (8.93% vs. 4.82%, p<0.001), and a higher rate of positive lymph nodes (56.22% vs. 48.64%, p = 0.001). Additionally, early-onset EC patients who underwent surgery were more likely to receive chemotherapy (75.13% vs. 61.91%, p<0.001) and radiotherapy (68.83% vs. 57.04%, p<0.001). There were no statistically significant differences (p>0.05) between early-onset and late-onset EC groups in terms of sex, histological type, and tumor grade. To reduce bias, we performed PSM at a 1:1 ratio, creating a matched cohort of 1,114 patients. After matching, there were no significant differences in the variables between the two groups (p>0.05).

**Table 1:** Demographic and clinicopathological characteristics of patients in early-onset and later-onset groups before and after propensity score matching (chi square test).

		Before PSM			After PSM		
		Early-onset	Later-onset	*p*	Early-onset	Later-onset	*p*
		n = 571	n = 4749		n = 557	n = 557	
**Sex (%)**	Female	95 (16.64)	774 (16.30)	0.883	89 (15.98)	91 (16.34)	0.935
	Male	476 (83.36)	3975 (83.70)		468 (84.02)	466 (83.66)	
**Race (%)**	Black	43 (7.53)	241 (5.07)	0.001	36 (6.46)	34 (6.10)	0.647
	White	487 (85.29)	4292 (90.38)		484 (86.89)	493 (88.51)	
	Others	41 (7.18)	216 (4.55)		37 (6.64)	30 (5.39)	
**Marital status(%)**	Married	344 (60.25)	3246 (68.35)	<0.001	339 (60.86)	332 (59.61)	0.713
	Unmarried	227 (39.75)	1503 (31.65)		218 (39.14)	225 (40.39)	
**Primary site (%)**	Upper	7 (1.23)	123 (2.59)	0.001	7 (1.26)	9 (1.62)	0.800
	Middle	53 (9.28)	603 (12.70)		53 (9.52)	48 (8.62)	
	Lower	465 (81.44)	3595 (75.70)		451 (80.97)	460 (82.59)	
	Others	46 (8.06)	428 (9.01)		46 (8.26)	40 (7.18)	
**Histology (%)**	EAC	418 (73.20)	3402 (71.64)	0.134	410 (73.61)	413 (74.15)	0.673
	ESCC	101 (17.69)	986 (20.76)		99 (17.77)	90 (16.16)	
	Others	52 (9.11)	361 (7.60)		48 (8.62)	54 (9.69)	
**Tumor size (%)**	<16mm	85 (14.89)	943 (19.86)	<0.001	83 (14.90)	73 (13.11)	0.425
	16-50mm	310 (54.29)	2651 (55.82)		306 (54.94)	327 (58.71)	
	>50mm	176 (30.82)	1155 (24.32)		168 (30.16)	157 (28.19)	
**Tumor grade(%)**	Grade I	52 (9.11)	373 (7.85)	0.821	48 (8.62)	50 (8.98)	0.989
	Grade II	232 (40.63)	1961 (41.29)		231 (41.47)	231 (41.47)	
	Grade III	233 (40.81)	1941 (40.87)		228 (40.93)	227 (40.75)	
	Grade IV	9 (1.58)	65 (1.37)		9 (1.62)	7 (1.26)	
	Unknown	45 (7.88)	409 (8.61)		41 (7.36)	42 (7.54)	
**T stage(%)**	T1	146 (25.57)	1501 (31.61)	<0.001	145 (26.03)	140 (25.13)	0.985
	T2	84 (14.71)	762 (16.05)		81 (14.54)	84 (15.08)	
	T3	290 (50.79)	2257 (47.53)		285 (51.17)	287 (51.53)	
	T4	51 (8.93)	229 (4.82)		46 (8.26)	46 (8.26)	
**N stage (%)**	N0	250 (43.78)	2439 (51.36)	0.001	245 (43.99)	249 (44.70)	0.856
	N1	321 (56.22)	2310 (48.64)		312 (56.01)	308 (55.30)	
**Chemotherapy (%)**	No	142 (24.87)	1809 (38.09)	<0.001	141 (25.31)	143 (25.67)	0.945
	Yes	429 (75.13)	2940 (61.91)		416 (74.69)	414 (74.33)	
**Radiation (%)**	No	178 (31.17)	2040 (42.96)	<0.001	175 (31.42)	174 (31.24)	1.000
	Yes	393 (68.83)	2709 (57.04)		382 (68.58)	383 (68.76)	

PSM:propensity score matching; EAC:esophageal adenocarcinoma; ESCC:esophageal squamous cell carcinoma.

### Comparison of survival outcomes between the early-onset and late-onset groups

Before PSM, the 1-year, 3-year, and 5-year OS rates were 84.7%, 60.9%, and 47.4% for the early-onset group, and 79.6%, 53.2%, and 42.4% for the late-onset EC patient group, respectively. Univariate Cox analysis showed significant associations of age, sex, race, marital status, histological type, tumor size, T stage, N stage, chemotherapy, and radiotherapy with OS(p<0.05). Further multivariate Cox regression including these variables identified age as an independent prognostic factor for surgical-treated EC patients, with early-onset EC demonstrating better OS (HR = 0.732, 95% CI: 0.655–0.819, p<0.001), as detailed in Table 2.

**Table 2:** Univariate and multivariate Cox regression analysis before propensity score matching.

		Univariate		Multivariate	
Variables		HR [95% CI]	*p*	HR [95% CI]	*p*
**Age**	Later-onset	Reference		Reference	
	Early-onset	0.774 [0.693–0.864]	<0.001	0.732 [0.655, 0.819]	<0.001
**Sex**	Female	Reference		Reference	
	Male	1.135 [1.039–1.241]	0.005	1.251 [1.138, 1.375]	<0.001
**Race**	Black	Reference		Reference	
	White	0.779 [0.681–0.892]	<0.001	0.895 [0.775, 1.034]	0.132
	Others	0.707 [0.579–0.863]	0.001	0.750 [0.613, 0.917]	0.005
**Marital status**	Married	Reference		Reference	
	Unmarried	1.135 [1.060–1.214]	<0.001	1.187 [1.107, 1.272]	<0.001
**Histology**	EAC	Reference		Reference	
	ESCC	1.280[1.184–1.383]	<0.001	1.242 [1.137, 1.356]	<0.001
	Others	1.285 [1.140–1.447]	<0.001	1.133 [1.004, 1.279]	0.044
**Primary site**	Upper	Reference			
	Middle	0.956 [0.770–1.187]	0.684		
	Lower	0.811 [0.663–0.991]	0.040		
	Others	0.883 [0.706–1.105]	0.277		
**Tumor size(mm)**	<16	Reference		Reference	
	16–50	1.769 [1.610–1.943]	<0.001	1.248 [1.125, 1.385]	<0.001
	>50	2.19 0[1.974–2.430]	<0.001	1.386 [1.229, 1.563]	<0.001
**Tumor grade**	Grade I	Reference		Reference	
	Grade II	1.355 [1.185–1.550]	<0.001	1.158 [1.011, 1.327]	0.035
	Grade III	1.781 [1.559–2.036]	<0.001	1.373 [1.196, 1.576]	<0.001
	Grade IV	1.914 [1.438–2.547]	<0.001	1.606 [1.204, 2.143]	0.001
	Unknown	1.076 [0.906–1.279]	0.402	0.967 [0.812, 1.151]	0.707
**T stage**	T1	Reference		Reference	
	T2	1.462 [1.317–1.622]	<0.001	1.376 [1.227, 1.542]	<0.001
	T3	2.201 [2.035–2.382]	<0.001	1.930 [1.739, 2.142]	<0.001
	T4	2.528 [2.190–2.919]	<0.001	2.287 [1.946, 2.687]	<0.001
**N stage**	N0	Reference		Reference	
	N1	1.875 [1.757–2.001]	<0.001	1.595 [1.477, 1.722]	<0.001
**Radiation**	No	Reference		Reference	
	Yes	1.312 [1.228–1.401]	<0.001	1.053 [0.936, 1.184]	0.391
**Chemotherapy**	No	Reference		Reference	
	Yes	1.287 [1.203–1.377]	<0.001	0.619 [0.545, 0.703]	<0.001

EAC:esophageal adenocarcinoma; ESCC:esophageal squamous cell carcinoma; HR:hazard ratio; CI:confidence interval.

To mitigate bias due to differences in variable distribution, we conducted further analysis after PSM. Post-PSM, the 1-year, 3-year, and 5-year OS rates were 85.2%, 58.4%, and 47.7% for the early-onset group, and 78.2%, 49.8%, and 39.1% for the late-onset EC patient group, respectively. Univariate Cox regression analysis indicated significant associations of age, tumor size, tumor grade, T stage, N stage, chemotherapy, and radiotherapy with OS in EC patients(p<0.05). Further multivariate Cox regression confirmed age as an independent prognostic factor for surgically treated EC patients, showing better OS for early-onset EC compared to late-onset EC (HR = 0.728, 95% CI: 0.630–0.842, p<0.001), as detailed in Table 3.

**Table 3:** Univariate and multivariate Cox regression analysis after propensity score matching.

		Univariate		Multivariate	
Variables		HR [95% CI]	*p*	HR [95% CI]	*p*
**Age**	Later-onset	Reference		Reference	
	Early-onset	0.739 [0.639–0.854]	<0.001	0.728 [0.630, 0.842]	<0.001
**Sex**	Female	Reference			
	Male	1.088 [0.889–1.331]	0.414		
**Race**	Black	Reference			
	White	0.772 [0.583–1.021]	0.070		
	Others	0.765 [0.513–1.141]	0.188		
**Marital status**	Married	Reference			
	Unmarried	1.152 [0.995–1.334]	0.058		
**Histology**	EAC	Reference			
	ESCC	1.177 [0.974–1.422]	0.092		
	Others	1.260 [0.984–1.615]	0.067		
**Primary site**	Upper	Reference			
	Middle	0.905 [0.478–1.715]	0.759		
	Lower	0.934 [0.514–1.695]	0.821		
	Others	1.033 [0.542–1.970]	0.922		
**Tumor size(mm)**	<16	Reference		Reference	
	16–50	1.754 [1.37–02.246]	<0.001	1.365 [1.048, 1.778]	0.021
	>50	2.296 [1.769–2.980]	<0.001	1.731 [1.295, 2.314]	<0.001
**Tumor grade**	Grade I	Reference		Reference	
	Grade II	1.286 [0.962–1.719]	0.089	1.104 [0.822, 1.482]	0.511
	Grade III	1.685 [1.264–2.246]	<0.001	1.402 [1.043, 1.883]	0.025
	Grade IV	2.925 [1.650–5.186]	<0.001	2.019 [1.128, 3.615]	0.018
	Unknown	0.899 [0.603–1.343]	0.604	0.736 [0.490, 1.104]	0.138
**T stage**	T1	Reference		Reference	
	T2	1.383 [1.075–1.779]	0.012	1.193 [0.907, 1.570]	0.207
	T3	1.954 [1.616–2.363]	<0.001	1.543 [1.216, 1.959]	<0.001
	T4	2.351 [1.773–3.117]	<0.001	1.978 [1.432, 2.731]	<0.001
**N stage**	N0	Reference		Reference	
	N1	1.914 [1.647–2.224]	<0.001	1.788 [1.501, 2.129]	<0.001
**Radiation**	No	Reference		Reference	
	Yes	1.276 [1.088–1.497]	0.003	0.884 [0.682, 1.147]	0.354
**Chemotherapy**	No	Reference		Reference	
	Yes	1.345 [1.131–1.600]	0.001	0.723 [0.537, 0.975]	0.033

EAC:esophageal adenocarcinoma; ESCC:esophageal squamous cell carcinoma; HR:hazard ratio; CI:confidence interval.

### Subgroup analysis after the PSM

**Fig 1:**
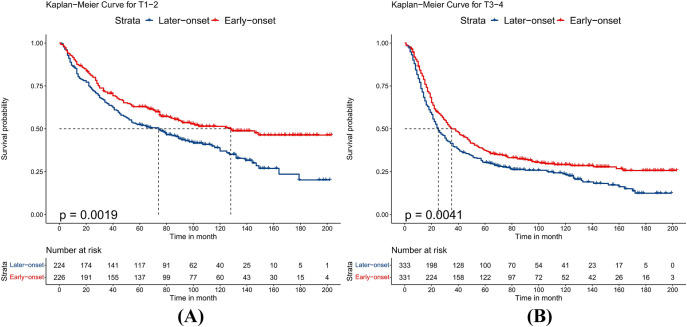
Kaplan–Meier curves for overall survival between early-onset and late-onset esophageal cancer. A.T1-2, B.T3-4.

**Fig 2:**
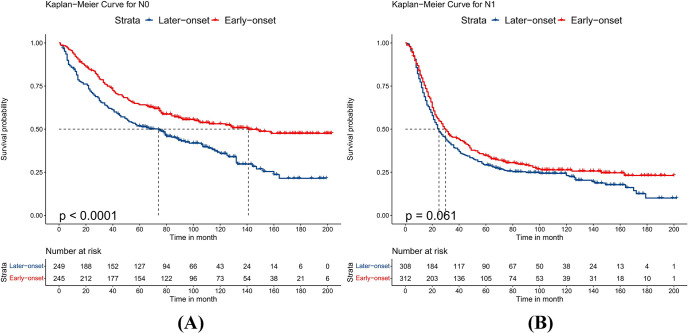
Kaplan–Meier curves for overall survival between early-onset and late-onset esophageal cancer. A.N0, B.N1.

**Fig 3:**
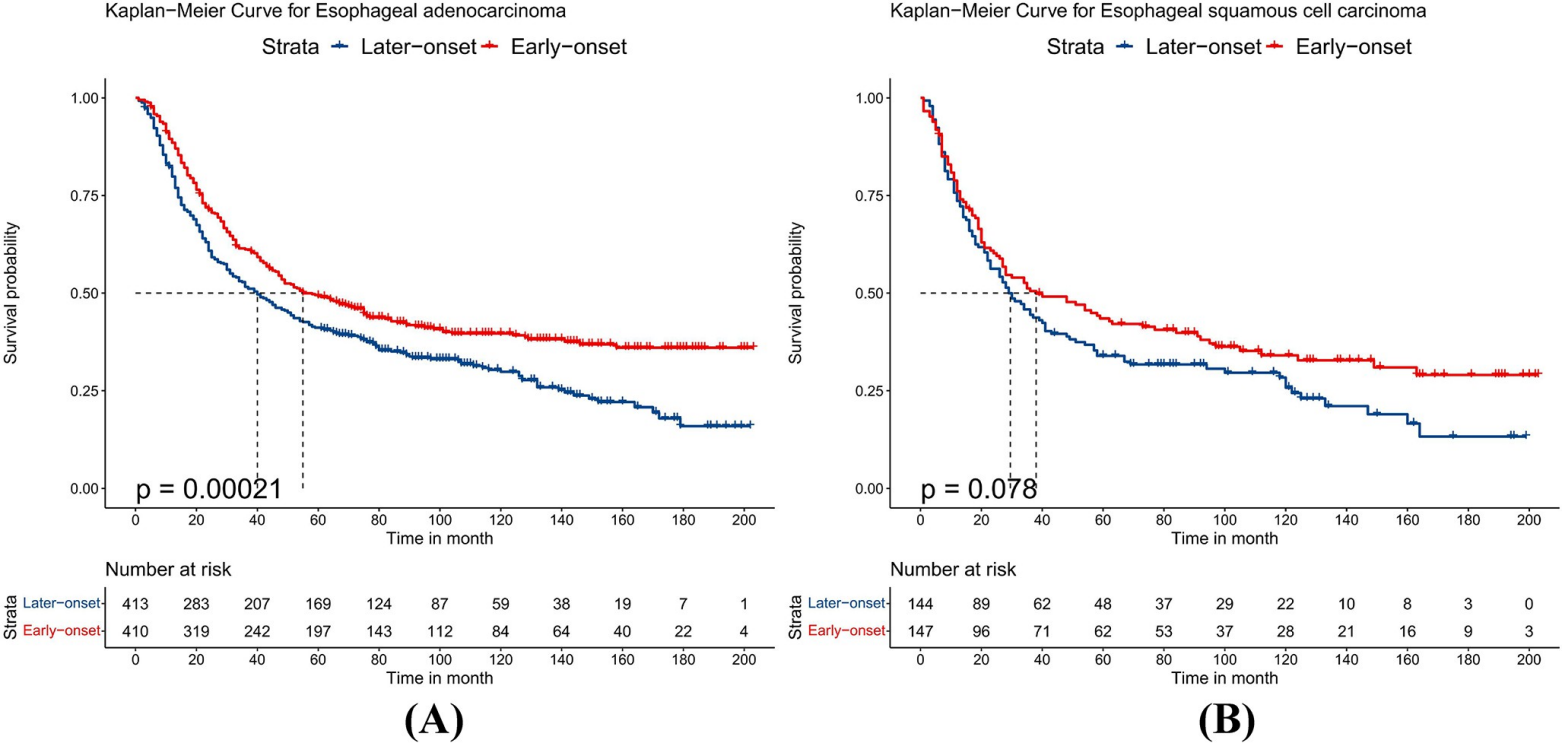
Kaplan–Meier curves for overall survival between early-onset and late-onset esophageal cancer. A. Esophageal adenocarcinoma, B. Esophageal squamous cell carcinoma.

**Fig 4:**
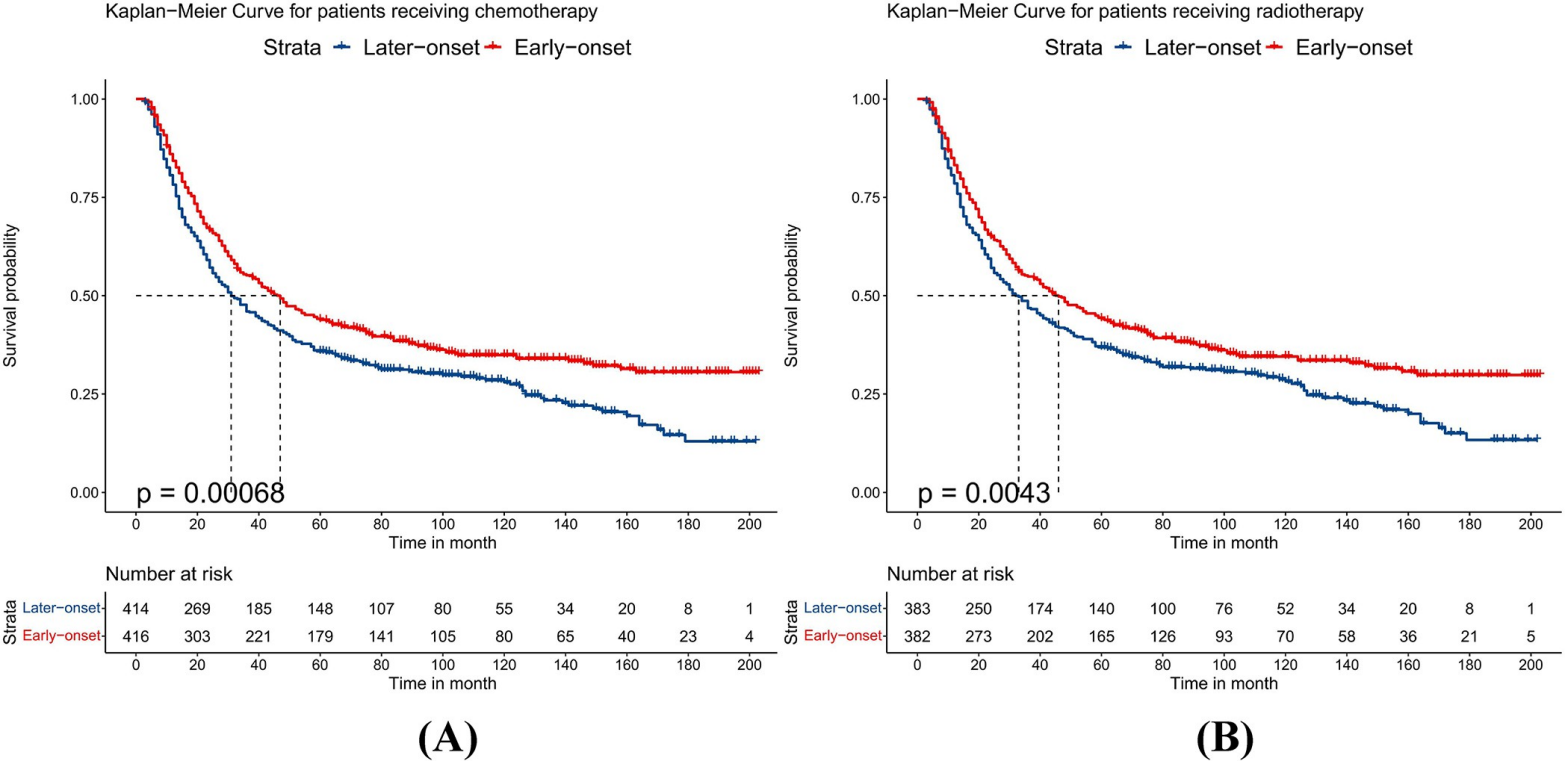
Kaplan–Meier curves for overall survival between early-onset and late-onset esophageal cancer. A. receiving chemotherapy, B. receiving radiotherapy.

Because the sample size of T2 and T4 stage patients was small after PSM, we combined patients into T1-2 and T3-4 groups to avoid bias. After PSM, the baseline characteristics of all subgroups were balanced. Compared to patients with late-onset EC, patients with early-onset EC had better OS whether their T stage was T1-2 or T3-4 (p<0.05), as shown in Fig 1. Stratifying by different N stages, patients with early-onset EC had better OS when the N stage was N0 (p<0.001). When the N stage was N1, there was no significant difference in prognosis between the two groups (p = 0.061), but the early-onset group showed a trend towards improved long-term survival, as detailed in Fig 2. For different histological types, patients with early-onset EAC had better prognosis than those with late-onset EAC (p<0.001). However, when the histological type was ESCC, there was no statistical difference in OS between the two groups (p = 0.078), although early-onset ESCC showed a trend towards improved long-term survival, as shown in Fig 3. Additionally, compared to late-onset EC, early-onset EC showed better OS when receiving chemotherapy (p<0.001) or radiotherapy (p = 0.004), as detailed in Fig 4. Although the survival differences between early-onset and late-onset EC patients were not statistically significant in the N1 (p = 0.061) and ESCC (p = 0.078) subgroups, their p-values were close to 0.05. Kaplan-Meier curves showed that the early-onset group had better OS than the late-onset group over the long term.

## Discussion

To our knowledge, this is the first large-sample study analyzing clinical characteristics and survival differences between surgically treated non-metastatic early-onset EC and late-onset EC. We found that early-onset EC had more severe tumor infiltration and more lymph node involvement compared to late-onset EC, but they received more aggressive radiotherapy and chemotherapy. Considering that differences in variables such as staging and treatment methods may lead to inaccurate prognostic analysis, we used PSM for precise assessment. The results showed that whether before or after PSM, patients with early-onset EC had better OS. Subgroup analysis after PSM revealed that early-onset EC patients generally had better outcomes compared to late-onset EC patients. In subgroups where there was no statistical difference in OS, the early-onset group also showed a trend toward improved long-term survival.

According to an analysis based on the SEER database, the annual incidence of early-onset EAC increased more than threefold from 1975 to 2015 [15]. A recent study based on the Global Burden of Disease (GBD) found that from 2010 to 2019, the incidence, death rates, and disability-adjusted life years of early-onset EC showed a declining trend in most regions globally [8]. This decline could be due to more focus on risk screening and advances in treatment technologies, though more research is needed. Nevertheless, early-onset EC remains a cancer with high mortality rates. Our study found that although the 5-year OS rate is higher than that of late-onset EC, it remains below 50%.

Our study found that early-onset EC patients present with more aggressive characteristics at initial diagnosis, attributed to several factors. Delayed diagnosis may be one reason. A study found that warning symptoms such as weight loss (40% vs. 57%, P = 0.0003) and dysphagia (29% vs. 42%, P = 0.0067) were less commonly observed in early-onset esophageal cancer patients [6]. For EC or gastroesophageal junction cancer patients, the median time to diagnosis was longer for early-onset cases (100 days vs. 64 days, p = 0.02), which may lead to later staging at diagnosis for early-onset patients [6]. Guidelines recommend endoscopic screening for Barrett’s esophagus (BE) patients with chronic reflux symptoms and three or more risk factors, including male gender, age >50 years, Caucasian ethnicity, smoking, obesity, and a family history of BE or EAC [16]. While the precursors of ESCC are complex, it is certain that both EAC and ESCC screening lack emphasis on younger populations [17].

Marital status is one of the socioeconomic factors, and being married can serve as a surrogate indicator of social support. Our study found that fewer early-onset EC patients were married compared to late-onset EC patients (60.25% vs. 68.35%, p<0.001). Research by Krajc et al demonstrated that married cancer patients are less likely to have metastases and have a lower likelihood of cancer-related mortality compared to unmarried patients [18]. Aizer et al similarly found that married patients have a lower likelihood of distant tumor metastasis and are more likely to receive curative treatment [19]. Additionally, research by Torrejon et al. found that early-onset EC patients have a higher proportion of non-white individuals and lower income, which aligns with our study findings. However, further research is needed to determine whether these differences are due to racial disparities or differences in socioeconomic status [20].

Our study found that early-onset EC patients who had surgery had better survival outcomes than late-onset EC patients, which is consistent with previous studies [12, 14, 21]. In contrast, Markar et al suggest that there is no difference in OS among EC patients of different age groups, possibly due to insufficient sample size [13]. However, prognosis in EC is not only associated with demographic and tumor characteristics but also closely linked to treatment modalities. Our data shows that early-onset EC patients are more likely to receive chemotherapy (75.13% vs. 61.91%, p<0.001) and radiotherapy (68.83% vs. 57.04%, p<0.001). Similarly, previous studies have shown that early-onset EC and late-onset EC patients receive different treatments, including radiotherapy and chemotherapy. These treatment differences may explain the variation in prognosis between the two groups. Additionally, our study found significant differences in race, marital status, tumor size, T stage, N stage, and the distribution of radiotherapy and chemotherapy between early-onset and late-onset EC patients (p<0.05). Cox regression analysis showed that these variables, as risk factors, influence prognosis to varying degrees. To determine whether differences in prognosis between early-onset and late-onset EC are due to the type of EC itself (early or late-onset), we applied PSM. After PSM, there were no significant differences in demographic, tumor characteristics, or treatment distribution between the two groups. Even post-PSM, early-onset EC patients still had a better prognosis than late-onset EC patients. This suggests that after accounting for staging, chemotherapy, radiotherapy, and other factors, early-onset EC patients still have better overall survival.

Apart from demographic characteristics, tumor features, and treatment methods, many factors contribute to the better prognosis of early-onset EC patients who undergo surgical treatment compared to late-onset EC patients. Early mortality and postoperative complications are important factors that must be considered. Markar et al.’s review found that elderly EC patients have higher risks after esophagectomy, compared to younger patients undergoing esophagectomy, elderly patients have increased in-hospital mortality (7.83% vs. 4.21%), along with higher rates of respiratory (21.77% vs. 19.49%) and cardiovascular complications (18.7% vs. 13.17%) [22]. The 5-year OS rate (21.23% vs. 29.01%, p<0.05) and 5-year disease-free survival rate (34.4% vs. 41.8%, p<0.05) are lower in the elderly group than in younger patients [22]. Mantziari et al conducted a more precise analysis, which also showed that compared to younger patients undergoing esophagectomy, elderly patients had higher rates of respiratory complications (20% vs. 16%), cardiovascular complications (15.6% vs. 7.0%), and postoperative mortality (7.9% vs. 3.4%) [23]. Additionally, compared to late-onset EC patients, early-onset EC patients have fewer comorbidities, better Performance Status (PS), and stronger physiological reserves and tolerance to stress [24, 25]. In some studies, age is considered a significant factor in competitive mortality, with advancing age, elderly patients are more likely than younger patients to die from heart failure, respiratory failure, stroke, falls, and other causes [26–28].

This study has limitations. Firstly, while this study included adjuvant therapies in patient prognosis analysis, the SEER database lacks specific chemotherapy regimens and radiotherapy doses. Additionally, information on prognosis-related factors such as PS, postoperative complications, and nutritional status is also lacking. Secondly, this study included patients diagnosed between 2004 and 2015, during which the American Joint Committee on Cancer (AJCC) sixth edition TNM staging system was used. Consequently, patients were categorized only as N0 and N1, and the guidelines at that time differed from recent advancements. The effect of these factors on our results is unclear and needs further investigation. Lastly, this study is based on large-scale retrospective analysis and lacks randomization. Although PSM was employed to minimize confounding factors, prospective data are still needed to further validate our conclusions.

In conclusion, early-onset EC patients without distant metastasis who undergo surgical treatment exhibit better OS outcomes, as confirmed by regression analysis and subgroup analysis conducted in matched cohorts.

## Supporting information

S1 Data The dataset used in this study. (XLSX)
